# Acknowledging extraordinary women in the history of medical entomology

**DOI:** 10.1186/s13071-022-05234-6

**Published:** 2022-03-31

**Authors:** Adriana Troyo, María Paula González-Sequeira, Mónica Aguirre-Salazar, Ian Cambronero-Ortíz, Luis Enrique Chaves-González, María José Mejías-Alpízar, Kendall Alvarado-Molina, Ólger Calderón-Arguedas, Diana Rojas-Araya

**Affiliations:** Laboratorio de Investigación en Vectores, Centro de Investigación en Enfermedades Tropicales, Sección de Entomología Médica, Facultad de Microbiología, Universidad de Costa Rica, San Jose, Costa Rica

**Keywords:** Entomology, Women, Science, Medical entomology, Scientist, Gender inequity, Arthropod

## Abstract

Throughout history, women have been actively involved in the advancement of science, while struggling to overcome challenges to participate and a lack of recognition. Prior to 1950, most women were not included in the lists of “classical” descriptions of the iconic scientific figures nor included in the most relevant historical accounts. Since the second half of the twentieth century, great efforts have been made to recognize the contributions of women to the advancement of science, especially since formal scientific careers have been dominated by men, with limited (or no) access to women. Despite these challenging social, political and cultural contexts, many women have succeeded in making significant advancements, and their contributions are now being acknowledged. Such efforts have led to the publication of recent reviews and compilations on outstanding women in biological sciences. The field of medical entomology is inherently interdisciplinary, focusing on insects and other arthropods that affect human health, with input primarily from the biological and medical sciences and a strong public health perspective. Several reviews and book chapters describing the history of medical entomology have been published over the decades, but few women are mentioned in these publications, even though many women have contributed to this field. Much of the information on these women is currently scattered throughout the published literature and historical records on a wide range of topics, including activism, virology, vector control and even acarology. Considering that there is no single available compilation of women contributors in the history of medical entomology, this review aims to provide a list of 22 women and their contributions to this field. The list includes women from diverse backgrounds, born in the late 1800s and before 1950, who directly impacted medical entomology in various ways and in different regions of the world. This compilation is far from exhaustive, but it aims to identify role models and examples of extraordinary women to motivate the evolving future of this field.

**Graphical Abstract:**

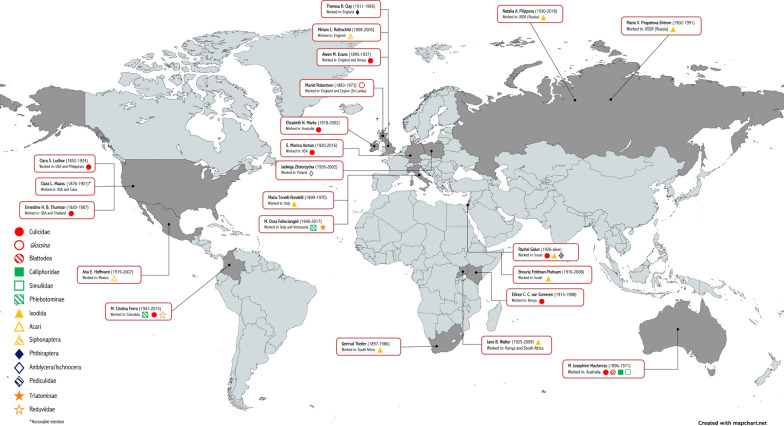

## Background

Women have been contributing to scientific developments since the beginning of science, although this has not always been evident. Even the most iconic female scientists in history, such as Marie Curie, Rosalind Franklin and Barbara McClintock, have had to struggle to be able to work in their fields and be acknowledged [1, 2]. Actually, it was not until the twentieth century that most universities and scientific academies formally allowed women to be included [1, 2]. In recent times, and especially in the last three decades, efforts have been made worldwide to understand the important roles played by women in science and reduce the gender gap. However, inequity between men and women in science remains a reality, and the vindication of the role of women in society has been variable and sometimes limited at high professional and academic ranks [3–9]. In addition, gender bias remains, and it can commonly be unintentional and unconscious [5, 10]. Studies show that one approach to overcome this bias is counter-stereotype imaging, such as showing women in roles where they have not usually been portrayed [11, 12]. Providing access to inspirational role models is also among the many other strategies to overcome the barriers facing women active in advanced scientific careers [13]. As a result, there are currently available many accounts and biographies of pioneering women who have influenced the early history of a wide range of scientific disciplines, including biological sciences [14–16].

From an academic point of view, modern medical entomology began in 1877–1878, with Patrick Manson’s discovery of the vector role of *Culex pipiens fatigans* in the transmission of *Wuchereria bancrofti* [17]. However, medical entomology, as a discipline, has only been recognized since the early 1900s and encompasses the study of insects and other arthropods that cause or transmit diseases that affect humans and other animals, with the direct purpose of preventing and controlling these afflictions [18–21]. As such, it is intrinsically interdisciplinary, with input from diverse areas, such as human and veterinary medicine, entomology, ecology, epidemiology, public health, microbiology, geography, molecular biology and social sciences [18, 20]. This concept has evolved and specialized throughout the years, and there are now different fields that have derived from the concept of medical entomology or have developed in close association with it, such as public health entomology, veterinary entomology, urban entomology, vector control, vector biology, vector ecology, among others [18, 22–24]. As with other scientific disciplines, the majority of the most relevant textbooks and reviews that describe the “golden age” of medical entomology summarize paramount discoveries made by notable men, such as Patrick Manson, Ronald Ross, Carlos Finlay, Walter Reed, Carlos Chagas, David Bruce, among others, but no women [17]. While it is not surprising that women were not involved in such famous discoveries given the social, political, and cultural circumstances of the time, a number of women have recently been identified whose contributions have been significant in the context of the developing field of medical entomology. Due to the multidisciplinary nature of this discipline, biographies and achievements of these prominent women are scattered, and there is currently no single document that compiles a list of these women who were pioneers specifically in medical entomology. This is unfortunate, considering that reading about the role of women who have been successful in a particular field can positively impact other women’s accomplishments [25].

In an effort to inspire current and future medical entomologists and raise awareness of the achievements of women in this discipline, this review highlights the life and main contributions of 22 women, all born before 1950, who have influenced medical entomology in various ways and in different parts of the world. This list is far from exhaustive and was initially constructed through web searches (using combinations of search items like “women,” “medical entomology,” “health,” “entomology,” “scientists,” “insect biology,” “vector biology”) and by receiving names from an open forum discussion among experts in entomology, medical entomology and other associated disciplines, which was initiated by the authors of this review. Subsequently, each name was investigated (available biographies, obituaries, scientific publications) to produce the final list presented here (see Table 1).

**Table 1:** Summary of information on selected women who have historically contributed to the development of medical entomology

Name	Years (birth–death)	Country of birth	Main countries of work	Most relevant arthropod group(s) (medical entomology)	Most relevant topic(s)
Clara S. Ludlow	1852–1924	USA	Philippines, USA	Culicidae	Taxonomy
Muriel Robertson	1883–1973	Scotland	England, Ceylon (Sri Lanka)	*Glossina*	Pathogen life cycle
Alwen M. Evans	1895–1937	England	England, Kenya	Culicidae	Taxonomy, ecology
M. Josephine Mackerras	1896–1971	Australia	Australia	Blattodea, Culicidae, Calliphoridae, Simuliidae	Taxonomy, epidemiology
Gertrud Theiler	1897–1986	South Africa	South Africa	Ixodida	Taxonomy, ecology
Maria Tonelli-Rondelli	1899–1970	Italy	Italy	Ixodida	Taxonomy
Maria V. Pospelova-Shtrom	1902–1991	USSR (Russia)	USSR (Russia)	Ixodida	Taxonomy, epidemiology
Miriam L. Rothschild	1908–2005	England	England	Siphonaptera	Anatomy, physiology, ecology
Theresa R. Clay	1911–1995	England	England	Phthiraptera	Taxonomy, ecology
Ellinor C. C. van Someren	1915–1988	Uganda	Kenya	Culicidae	Taxonomy Ecology
Brouria Feldman-Muhsam	1916–2008	Palestine (Israel)	Israel	Ixodida	Physiology, ecology
Elizabeth N. Marks	1918–2002	Ireland	Australia	Culicidae	Taxonomy
Ana E. Hoffmann	1919–2007	México	México	Acari	Taxonomy, biology
Ernestine H. B. Thurman	1920–1987	USA	Thailand, USA	Culicidae	Taxonomy, ecology, control
S. Monica Asman	1920–2016	Germany	USA	Culicidae	Control, genetics
Jane B. Walker	1925–2009	Kenya	Kenya, South Africa	Ixodida	Taxonomy, biology
Jadwiga Złotorzycka	1926–2002	Poland	Poland	Amblycera /Ischnocera	Taxonomy, ecology, morphology
Rachel Galun	1926-alive	Palestine (Israel)	Israel	Culicidae, Ixodida, Pediculidae	Physiology, ecology
Natalia A. Filippova	1930–2018	USSR (Russia)	USSR (Russia)	Ixodida	Taxonomy, epidemiology
M. Dora Feliciangeli	1940–2017	Italy	Venezuela, Italy	Triatominae, Phlebotominae	Epidemiology, biology, genetics, taxonomy
M. Cristina Ferro	1947–2015	Colombia	Colombia	Phlebotominae, Culicidae, Reduviidae	Taxonomy, biology, ecology, epidemiology
Clara L. Maass^a^	1876–1901	USA	USA, Cuba	Not applicable	Died in yellow fever transmission experiments

^a^Honorable mention

## Clara Southmayd Ludlow

Clara Southmayd Ludlow was born in 1852 in Pennsylvania, USA [26]. At first, she showed interest in music and graduated from the New England Conservatory of Music in 1879; later, she became interested in science [26]. From 1897 to 1900, she attended the Mississippi Agricultural and Mechanical College, where her interest in mosquitoes began under the tutelage of George Herrick, a biology professor [26]. It was George Herrick who first introduced her to medical entomology, working in a yellow fever (YF) laboratory [27]. At the Mississippi Agricultural and Mechanical College, she subsequently obtained a Bachelor of Science (B.S.) degree in agriculture and then a Master of Science (M.Sc.) in botany [27]. After graduating in 1901, she traveled to visit her brother, an officer working with the US Army Forces in the Philippines. Here, she met Dr. William Calvert, who was amazed by her interest in mosquitoes and by her work at Herrick's YF laboratory and consequently convinced Colonel B.F. Pope to issue an order that allowed medical officers to collect mosquito specimens and send them to Manila for further examination by Dr. Ludlow [27]. She returned to the USA that same year, and in 1904 she moved to Washington DC, and started working at the Army Medical Museum [28]. Some years later, in 1908, she received her Doctor of Philosophy (Ph.D.) degree from George Washington University [26]. At this institution, not only was she a student, but also an instructor of histology and embryology until 1911 [28]. In 1920, Dr. Ludlow became the chief entomologist at the Army Medical Museum (now National Museum of Health and Medicine), a position she held until her death [29].

Dr. Ludlow became an expert in mosquito taxonomy and published around 53 scientific papers, 20 of which, all on mosquitoes, were published during the time she was at George Washington University. Some of her most relevant work focuses on the description of mosquito species. In 1905, she described the morphology and the differences between males and females of *Taeniorhynchus sierrensis* (now in the genus *Aedes*), collected from three different rivers throughout the USA [30]. She also described *Aedes albopictus* as *Stegomyia scutellaris* var. samarensis, not knowing that Frederick A. A. Skuse had already published a paper describing it as *Culex albopictus* [31]. However, the presence of this mosquito in the Hawaii islands was confirmed because of Dr. Ludlow’s studies [31]. She also described *Anopheles perplexens* in Pennsylvania [28] and worked on mosquitoes from the North and West Indies and the Philippine Islands, where she described those mosquitoes that serve as disease vectors, as well as the life-cycles and breeding preferences of different Culicinae and Anophelinae mosquitoes [32].

Dr. Ludlow was honored by her peers by becoming the first non-physician and woman member of the American Society of Tropical Medicine and Hygiene (ASTMH) in 1908. In 2016, the ASTMH council voted in favor of creating a medal named after an iconic leader in tropical medicine and created the “Clara Southmayd Ludlow Medal” in recognition of her work.

Dr. Clara Ludlow died of cancer in the USA in 1924 [26].

## Muriel Robertson

Muriel Robertson was born in Glasgow, Scotland in 1883, although she lived most of her professional life in London, England [33]. She was taught at home by a governess until she entered Glasgow University, and while she had no scientific schooling in her childhood, the home in which she grew up was permeated with the tints of science [33]. It was at this university that she received her first formal scientific education, and her interest in science was roused when she began studying zoology and botany. Under the influence of Professor Kerr, she started her first research projects as an undergraduate student, investigating the life-cycles of protozoans [33]. Her first article was published in 1905, and it described the life-cycle of *Pseudospora volvocis*, an organism that parasitizes colonies of *Volvox* algae [34]. That same year, she graduated from Glasgow University with a Master of Arts degree [33].

In 1907, Dr. Robertson moved to Ceylon (currently Sri Lanka) to study hemoparasites of reptiles, with an emphasis on trypanosomes and their life-cycles [35–39]. She returned to Glasgow in 1908 and moved to London in 1909 to work as an assistant at the Lister Institute of Preventive Medicine, where she became a staff member 1 year later. At the beginning of the twentieth century, Commissions under the auspices of the Royal Society were sent to study an epidemic of sleeping sickness in Uganda. Considering that she already had experience studying fish and reptile trypanosomes, Dr. Robertson was appointed as a Protozoologist in the Protectorate of Uganda in 1911. This assignment was remarkable, as others have stated in her biography: “The appointment at that period of a young woman to a post in tropical Africa was not only an indication of the reputation which her work upon trypanosomes had already earned, but of the opinion of the Appointing Committee of her strength of character and courage” [33]. In 1914, Dr. Robertson returned to the Lister Institute, where she remained until 1961 [33, 40].

During her time in Uganda, Dr. Robertson made notable contributions to medical entomology by investigating the development and life-cycle of *Trypanosoma gambiense* (now *T. brucei gambiense*) in *Glossina palpalis* (tsetse fly), as well as in the human blood, making path-breaking discoveries [41–44]. For example, she studied the fluctuations in numbers of trypanosomes present in the animal’s blood, the polymorphism of the parasites and how these factors influenced the infection outcome in *G. palpalis*. She also provided evidence that a specific ‘type’ of trypanosome was responsible for continuing the life-cycle in the invertebrate host. In addition, she determined factors that interfere with the infection of tsetse flies by the trypanosomes.

Dr. Robertson also contributed to many different topics of parasitology and bacteriology. She made significant advances in the study of the free-living ciliate *Bodo caudatus* [45–47], as well as in the immunology of trichomoniasis in cattle, including the development of a diagnostic agglutination test and studies on antibody responses in *Trichomonas foetus* infections [33, 40, 48–52]. Due to the increase in cases of bacterial gas gangrene in war wounds (World Wars I and II), she also studied bacterial agents, treatments and vaccines for these diseases [33, 40, 53–57].

Dr. Muriel Robertson was a remarkable parasitologist and bacteriologist, which was acknowledged in 1947 when she was elected as a Fellow of the Royal Society, becoming the eighth woman to have received this honor [33]. She was also awarded an Honorary Doctorate in Law from the University of Glasgow in 1948, and was recognized a fellow of the Royal Society of Tropical Medicine and the Institute of Biology. In addition, Dr. Robertson was a founding member of the Society for General Microbiology, being on its council from 1945 to 1948, and was declared an honorary member in 1962 [40]. She was also a member of the Medical Research Club, the Society for Experimental Biology, and the Pathological Society.

Although she spent most of her life in London, Dr. Muriel Robertson had a deeply rooted affection for Limavady in Northern Ireland, where she died in 1973 [33, 40].

## Alwen Myfanwy Evans

Alwen Myfanwy Evans was born in 1895, in Stockport, England, but lived most of her life in Mossley Hill, Liverpool. Dr. Evans earned a degree in entomology at Manchester University and started her outstanding scientific career in 1918, when she began working at Liverpool School of Tropical Medicine [58]. There, she got promoted, and by 1921, she became the school’s first female lecturer in the Department of Entomology, studying tsetse flies before specializing in tropical insects [59–61]. Five years later, she went on expeditions to African countries and, when she returned to England, Dr. Evans acquired her Doctor of Science (D.Sc.) from Manchester University for her thesis “A Short-Illustrated Guide to the Anophelines of Tropical and South Africa,” in which she provided a collected account of Ethiopian anophelines, their habits and breeding places [58, 62].

In terms of her achievements, Dr. Evans is known for specializing in mosquitoes, particularly African anophelines [63–66]. Starting with the results of her expeditions to Africa, she was able to undertake surveys and identify insects from Sierra Leone and Kenya [67–69]. Based on online research, there are approximately 35 documents, between notes and publications authored and co-authored by Dr. Evans; however, there is no certainty as to this total amount. Several of these documents describe *Anopheles marshalli* varieties and specimens belonging to the *Anopheles funestus* complex [70–76].

The descriptive drawings made by Dr. Evans distinguishes her academic career, since these exceptional illustrations are often used for educational/teaching purposes and continue to stun current students and researchers. Her drawings can be seen throughout all her work and are depicted in her world-famous monograph “*The Mosquitoes of the Ethiopian Region*”, which includes mosquito larval habits and breeding places, habits of the adults, associations with malaria, and distribution of the anophelines of the region [77]. Since anophelines are responsible for vectorial transmission of plasmodia, one of Dr. Evans’s goals was to establish a starting point for other researchers to discover control methods for mosquito-borne diseases (mentioned by A.M. Evans in an undated newspaper article provided by the Liverpool School of Tropical Medicine). In 1926, Jean Brèthes, described the species *Anopheles evansae* from Argentina, which was dedicated to Dr. Evans [78].

Dr. Alwen M. Evans lived a short life and died of pneumonia in 1937, just a few days after completing the monograph that includes the written records of all her life’s work, which was published a year later [58, 77].

## Mabel Josephine Mackerras

Mabel Josephine Mackerras was born Mabel Josephine Bancroft in 1896, in Queensland, Australia. Her father was Thomas Lane Bancroft, a physician and naturalist known for studying the life-cycle of filariae in their vectors [79, 80]. He introduced her to natural history as she used to help him prepare zoological specimens to send to the Queensland Museum [79, 80]. She obtained her B.S. from the University of Queensland in 1918 and then went on to study medicine at the University of Sydney, graduating in 1924 with a medical degree; both degrees were earned with honors [79]. In 1924 she married Ian Murray Mackerras, who had also graduated in medicine, and adopted his last name [80]. In her early career, she worked as an Assistant Entomologist in the Division of Economic Entomology of the Council for Scientific and Industrial Research in Canberra, Australia [80]. She resigned when World War II (WWII) broke out and became a Major in the 2nd Australian Imperial Force, specifically in the Medical Research Unit. After the war, she joined the Queensland Institute of Medical Research as a Senior Parasitologist, where she spent some of the most productive years of her career [80].

Dr. Mackerras authored and co-authored approximately 86 papers on several different topics, mostly within the Diptera and Blattodea, but also on hematozoan parasites of Australian vertebrates, nematodes in animals and diagnosis and prevention of salmonellosis in children [80]. Among some of her most relevant contributions in field of medical entomology are from her research on malaria and experimental infections in volunteer army personnel (during WWII) [80]. In these experiments, she bred adults of different species of Australasian anophelines, infected them with human malaria parasites and studied their susceptibility to infection and behavior in captivity, as well as the life-cycle of the parasite [79, 81, 82].

Regarding other Diptera, Dr. Mackerras also researched blowfly infestations in sheep, focused on the growth of the larvae of *Lucilia cuprina*,* Lucilia sericata* and *Chrysomya rufifacies,* and made observations regarding the life cycle of these species along with *Calliphora stygia* and *C. augur*, while experimentally breeding them [83, 84]. Also, in collaboration with her husband, she made revisional notes on the taxonomy of the family Simuliidae, focusing on the genera present in Oceania (*Cnephia *,* Simulium* and *Austrosimulium*) and describing two species (*Simulium torresianum* and *Austrosimulium magnum*) [85–88].

Dr. Mackerras also worked with the order Blattodea. She participated in writing a chapter about this group in the book *The insects of Australia* [80] and, as an Australian taxonomist, she described and redescribed approximately 14 different genera (*Anamesia*,* Cosmozosteria*,* Desmozosteria*,* Drymaplaneta*,* Euzosteria*,* Leptozosteria*,* Megazosteria*,* Melanozosteria*,* Platyzosteria*,* Polyzosteria*,* Pseudolampra*,* Scabina*,* Temnelytra* and *Zonioploca*), contributed taxonomic notes on various genera (*Austrostylopyga*,* Dorylaea*,* Eppertia*,* Methana* and *Tryonicus*) and made one of the most complete compilations of the Polyzosteriinae subfamily to date [89–98]. During an epidemic of salmonellosis, she also discovered the role of cockroaches as a source of the bacteria in hospital environments [79, 99].

The academic activity of Dr. Mackerras also included collaborations with other scientists, such as Professor T. Harvey Johnston, with whom she published several articles describing three new species of *Musca* and the pupae and larvae of three species of Tabanidae, and on bovine tick resistance [79, 80, 100]. Likewise, she played an important role in the collection and classification of mosquitoes to find the vector of the Murray Valley encephalitis virus, which finally led to the isolation of the virus from *Culex ermutirostris* specimens [101, 102].

Dr. Mabel Josephine Mackerras was awarded the D.Sc. *honoris causa* from the University of Queensland in 1967 [79]. She passed away in Canberra in 1971 [80].

## Gertrud Theiler

Gertrud Theiler was born in 1897 in Pretoria, South Africa. She was inspired by her father, Arnold Theiler, a famous veterinarian who worked with ticks and mites [103]. She obtained her B.S. degree from South African College and then focused on helminths during her Master’s studies at the University of Neuchâtel, Switzerland, and at the Liverpool School of Tropical Medicine and London School of Tropical Medicine, UK [104]. She returned to South Africa in 1924, where she first worked as a lecturer at Huguenot College and later, in 1939, as a lecturer at Rhodes University College; In 1940 she resigned from the latter institution and accepted a research position in Entomology at Onderstepoort, Pretoria [105].

Ms. Theiler’s research focused mainly on ticks and acarology. Due to the lack of clarity in the *Amblyomma marmorenum* group, she studied this genus of ticks in collaboration with Louis Salisbury and characterized different specimens collected as free-living stages or from multiple hosts, such as tortoises and pythons [106]. Her work also included an update of the description, host list and distribution of lesser known African *Rhipicephalus* species, such as *Rhipicephalus follis*, *R. sulcatus*, and others. Specimens of these species were collected from different African countries, including Rhodesia (now Zimbabwe), Uganda and South Africa [107]. In addition, she studied and made accurate distribution maps across Africa of different species of *Hyalomma* [108], as well as of of the cape brown tick, *Rhipicephalus capensis*, also specifying, for the latter, the type of forest, tick habits and how its distribution is affected by temperature and altitude [109]. Her interests also included host specificity, mostly on the ticks of birds. She organized and analyzed the records of specific associations for several adult ticks and African birds, such as *Argas persicus* with domestic fowl, but also included birds that act as intermediate or accidental hosts for some African ticks [110]. In time, her studies on host tick specificity not only expanded to other groups, such as mammals and reptiles, but also included adults, nymphs and larvae of different tick species, such as *Dermacentor rhinocerinos*, *Rhipicephalus sanguineus*, *Margaropus winthemi*, among others [111].

Ms. Theiler joined the staff of the Veterinary Research Institute, which was created by her father Arnold after his death, and helped in its development by sharing her findings on African ticks [104]. Unfortunately, she had to prematurely stop her studies and research due to loss of hearing and sight [105].

Ms. Gertrud Theiler died in Cape Town, South Africa in 1986, in the company of her close friend Andria van Gass, with whom she spent the last years of her life [104].

## Maria Tonelli-Rondelli

Maria Tonelli-Rondelli was born Maria Rondelli in 1899, in Turin, Italy. She studied natural sciences (1921) and geography (1923) at the University of Turin [112]. In 1927, she married Leonida Tonelli and changed her name to Maria Tonelli-Rondelli, a double surname that she used in all her scientific publications [112, 113]. After finishing her studies, she collaborated with the museums and institutes of geology and zoology of the University of Turin, and started her academic contributions by translating into Italian the volume *Zoology by Rémy Perrier*, written by the zoologist Umberto Pierantoni [112].

The main entomological contribution of Ms. Tonelli-Rondelli was the study of ticks (Ixodidae), especially South American species [114–117]. She studied the specimens described by Koch in 1844 [118] and published additional descriptions, redescriptions and illustrations of the genus *Amblyomma* [115]. She was one of the first researchers to propose that *Amblyomma cajennense* was a species complex [115], described new species of *Amblyomma*, such as *Amblyomma latepunctatum* and *Amblyomma romitii* [117] and verified the validity of species such as *Amblyomma mixtum*, *Amblyomma tenellum* and *Amblyomma sculptum* [115, 119]. However, in 1953, Aragão and Fonseca considered that her work was erroneous, declaring that the morphological characters used as differential criteria by Ms. Tonelli-Rondelli were due to intraspecific variability between specimens [115, 120, 121]. Consequently, all the taxonomic names given by Ms. Tonelli-Rondelli at that time were considered synonymous [121]. This debate continued and strengthened over the years since other researchers were not in favor of Ms. Tonelli-Rondelli's findings either; for example, Vogelsang and Santos Dias proposed that *Amblyomma aureolatum* (under the name *A. striatum*) was a synonym of *A. ovale* [119, 122].

In 2013, Beati and colleagues found molecular differences between the species included at that time in the *Amblyomma cajennense* group, separating them into six geographically defined groups. Their study validated the work of Ms. Tonelli-Rondelli, and the authors concluded that *A. cajennense* is a complex of species [123]. Today it is also accepted that *A. aureolatum* and *A. ovale* are different and valid species [119]. In 2014, Nava and collaborators confirmed some of her taxonomic hypotheses related to a selection of *Amblyomma* species and named a new tick species after her: *Amblyomma tonelliae* [121].

Ms. Maria Tonelli-Rondelli died in 1970 [112].

## Maria V. Pospelova-Shtrom

Maria V. Pospelova-Shtrom was born in imperial Russia in 1902 [124]. Her father was an entomologist and probably influenced her interest in this discipline [125, 126]. After obtaining her Ph.D. degree, she became the head of the Tick Laboratory in the Department of Medical Entomology at the Institute of Parasitology [124].

During her career, Dr. Pospelova-Shtrom worked mainly on tick-borne spirochetosis [127–129]. She coordinated 36 entomological surveys in Central Asia, Kazakhstan, the Caucasus and Central Russia to learn about the main aspects of the argasid (Ixodida: Argasidae) fauna of these regions. The entomological material collected was the basis for important studies related to the morphology, experimental and field ecology, taxonomy and systematics of argasid ticks. Her publication entitled* On the system of classification of ticks of the family Argasidae* is considered to be one of her most significant contributions [130]. Among other documents published by Dr. Pospelova-Shtrom is a book on the subfamily Ornithodorine and its epidemiological importance, which was translated into English and published in the USA by the US Department of Commerce. She also published four monographs that represent significant contributions to the field of acarology [125].

The taxonomy and systematics of ixodid ticks were also topics researched by Dr. Pospelova-Shtrom, and several of her publications were devoted to the genus *Haemaphysalis* [131, 132]. Additionally, she studied the experimental management of ticks, laboratory feeding conditions for research with ixodid ticks and the use of dichlorodiphenyltrichloroethane (DDT) as a possible agent for tick control [133, 134]. Regarding tick taxonomy, she described four new species: *Argas beklemischevi*, *Haemaphysalis erinacei turanica*, *Haemaphysalis pavlovskyi* and *Haemaphysalis pentalagi* [135].

In her role as an expert in the field of tick biology, Dr. Pospelova-Shtrom participated in other academic activities. For example, she organized, together with WHO, the First International Meeting on Ticks and Their Diseases, held in Switzerland. Likewise, her knowledge and experience allowed her to play an important role as a mentor and teacher by overseeing graduate students, resulting in around 20 graduate theses, including 10 doctoral dissertations [124].

In light of the importance of her contributions to the field of tick biology, she was honored with the dedication of the species *Haemaphysalis pospelovashtromae* [136]. Dr. Pospelova-Shtrom passed away in Kiev, Ukraine in 1991 [124, 125].

## Miriam Louisa Rothschild

Miriam Louisa Rothschild was born in 1908 in Northampton, England. She was tutored in natural history, but she did not have a traditional academic education. Nevertheless, for her outstanding research endeavours she was awarded honorary degrees by major European institutions, including D.Sc. from Oxford University (1968), Göteborg University (1983) and Cambridge University (1999) [137, 138]. Her drive for science was influenced by her father Charles Rothschild, a famous entomologist and conservationist [139].

Throughout her productive career, Dr. Rothschild published more than 300 scientific papers [138]. Although her research started with parasites of marine snails and seabirds, one of her major academic contributions to medical entomology was cataloging the Rothschild flea collection for over 30 years [140]. She created this collection as a legacy to her father, and it is currently located at the Natural History Museum in London [141]. This collection not only includes specimens obtained from different places and hosts, but also a database developed by Dr. Rothschild that includes information on the distribution, host preferences, sex and detailed structures of fleas, such as their eyes, ovaries and vagina [142]. She dissected and examined the internal structure of more than 600,000 sections of 32 species of fleas and found differences in specific organs or systems; for example, between the central nervous system in *Tunga monositus* males and that of other species, where the terminal portion of the ventral nerve cord becomes V-shaped and the terminal ganglion lies dorsally due to the development of the aedeagus [143]. The collection also includes 143 fleas (e.g. several *Ceratophyllus* species) from 36 species of birds, together with information on their distribution and host preferences, along with handmade illustrations of their geographical distribution [144]. Another one of her notable publications, which was coauthored by Dr. Theresa R. Clay, was the book *Fleas, Flukes & Cuckoos; A Study of Bird Parasites*, a text devoted to bird parasites and their relationships, arguably the first of its kind [145]. In addition, Dr. Rothschild is known for her research on the regulation of the rabbit flea’s life-cycle mediated by hormones of the vertebrate host [146, 147]. Moreover, one of her major contributions was the description of the jumping mechanism in *Xenopsylla cheopis*. This work included a series of images that showed the sequence of movements in the flea body leading up to its jump, their position, jump velocity and acceleration and notes about their jumping ability even if their entire back leg was cut [148].

Dr. Rothschild had other academic interests as well, including insect chemical ecology, and she was a pioneer in the extraction of chemical plant compounds [140]. She discovered, for example, that the European cinnabar moth, *Callimorpha jacobaeae*, can sequester a pyrrolizidine alkaloid from their host plant and retain it through all their metamorphosis [149]. She also demonstrated that *Zyganea flupendular* and *Z. lonicerae* (Lepidoptera) can release hydrocyanic acid [150]. Furthermore, Dr. Rothschild was involved in the paper that confirmed that some butterflies (*Danaus plexippus* and *D. chrysippus*) have cardenolids (i.e. a heart poison) in their bodies, making them indigestible and distasteful for most vertebrates [151].

In addition to the honorary degrees mentioned above, Dr. Rothschild received several recognitions and awards, including the Bloomer Award (Linnean Society), Wigglesworth Gold Medal (Rota Entomological Society), Victoria Medal of Honour (Royal Horticultural Society) and Mendel Award (Czech Science Academy). She was also named Fellow of the Royal Society and Dame Commander of the Order of the British Empire [138].

Dr. Miriam Rothschild died in 2005, in the same city where she was born 96 years earlier. At the time of her death, she was actively investigating the sequestering of toxic compounds from plants [140].

## Theresa Rachael Clay

 Theresa Rachael Clay was born in the UK in 1911 [152]. She grew up in Notting Hill, London, where she attended school [153, 154]. In her early scientific career in zoology, she spent the 1930s traveling with her cousin, Colonel Richard Meinertzhagen, who was an ornithologist. In these travels, Colonel Meinertzhagen would ask curators permission for her to gather lice (Mallophaga) from bird specimens in their collections [154]. Between 1935 and 1938 she went on expeditions to Africa, the Middle East and the Artic as an independent researcher. After WWII, she continued her expeditions (1946–1949), only now as an entomologist at the British Museum, which at that time included the current National History Museum; during this time she was volunteering in an unofficial capacity. In 1949 she became a temporary staff worker at the British Museum [153], followed in 1952 with the appointment as a senior scientific officer and then in 1955 as Principal of the Apterygota collection. That same year, 1955, she received the D.Sc. degree from the University of Edinburgh. In 1970 she was appointed Deputy Keeper of Entomology, a position she kept until her retirement in 1975. In 1975 she married Rodney G. Searight [153].

While working at the British Museum, overseeing the Phthiraptera and Apterygota sections, Dr. Clay became a world-renowned authority on lice. She collected specimens from several regions, such as India, Pakistan, the western Himalayas, Trinidad, British Guiana and Malaysia [153]. Altogether, she published about 40 papers, mainly on the chewing lice, including reviews on taxonomy and names, various studies on bird lice distribution, taxonomic keys to several mallophagan genera, species descriptions and species checklists [155–167]. Moreover, some of her publications involve sucking lice (Anoplura) of sciurids (*Neohaematopinus*) and pinnipeds (*Antarctophthirus*,* Lepidophthirus*), based on collections from her expeditions [165, 168]. Dr. Clay also coauthored *Fleas, Flukes & Cuckoos; A Study of Bird Parasites*, together with Miriam Rothschild [145]. Additionally, during her lifetime, she classified over 23 genera, adding to the development of mallophagan classification. Towards the end of her career, she worked on mammal lice as well [169].

To date, Dr. Clay’s most valuable contribution to medical entomology may be *A checklist of the genera & species of Mallophaga*, published in 1952, which she co-authored with George Hopkins [159]. Before this publication, other checklists had been published which had proven to be incomplete (see [170]). In her work, all publications regarding bird lice taxonomy were examined, with species being reclassified in a way that represented the opinion of most specialists on the subject at the time. It has been pointed out that this particular document marked the beginning of a new era in lice taxonomy and that the publication served as a basis for future research, with only minor changes at the time [169].

Records of Dr. Clay’s recognitions by her peers and contemporaries are available. One of these is Dr. K.C. Emerson’s *Lice in my life*, a work portraying his views on the evolution of lice studies, wherein he mentions the contributions of Dr. Clay, with whom he corresponded, collaborated and exchanged specimens, to the study of Phthiraptera taxonomy. He comments on her every paper as being “excellent and a real contribution” to the knowledge of chewing lice [169]. Another example of her merits can be found in George Hopkins’ work, *New African Mallophaga*, in which he describes a new genus and species, namely *Clayia theresae*, in honor of Dr. Clay and her life’s trajectory on bird lice [171]. This is not, however, the sole species dedicated to Clay, since K. C. Emerson described *Menopon clayae* based on material from the British Museum in 1954 [172]; 10 years later, he described *Chinchillophaga clayae*, with specimens provided by Dr. Clay herself [173]. Lastly, 2 years later, he and R.D. Price described *Kelerimenopon clayae*, with resources provided by Dr. Clay and the British Museum [174]. In the same manner, R. Meinertzhagen named the Afghan snow-finch, an authentic species also known as Theresa’s snow-finch (*Pyrgilauda theresae*), after her [154].

Dr. Theresa Clay Searight died in 1995, at Brecon House Nursing Home in Sherborne, Dorset, at the age of 84. Her life’s work is kept at the National History Museum [153, 175].

## Ellinor Catherine Cunningham van Someren

Ellinor Catherine Cunningham van Someren was born Ellinor Catherine MacDonald in the city of Kampala, Uganda in 1915 (official documentation kindly provided by Noel van Someren and mentioned in [176]). However, other records incorrectly mention 1916 and South Africa as the year and place of her birth, respectively [177, 178]. The only formal education received by Ms. van Someren was at Iverness Royal Academy in Scotland (documentation provided by N. van Someren). In 1940, she married G. Robert Cunningham van Someren, a well-known ornithologist who worked for the East Africa Research Unit in Kenya, after which she used only her married name [179]. In 1936, Ms. van Someren was appointed a Junior Laboratory Assistant in the Division of Insect-borne Diseases of the Medical Research Laboratory in Nairobi, Kenya, where she continued working until 1973 [176, 180]. Although Ms. van Someren did not have complete additional advanced studies or obtain academic degrees, her knowledge of mosquito taxonomy and biology is clearly evident from her numerous publications and activities, including his position as a consultant for WHO during and after the YF epidemics that occurred in 1962 (documents provided by N. van Someren).

Ms. van Someren was a renowned mosquito taxonomist, and her most important contributions focus on Culicidae of East Africa. One of her first publications (still under her maiden name) appeared in 1939, in which she describes the larvae of *Aedes* (*Finlaya*)* pulchrithorax* Edwards in detail, based on specimens collected in Nairobi [181]. In the mid-1940s and early 1950s, she published many descriptions as a single author, mostly in the series* Ethiopian Culicidae*,” where she included several new species, subspecies, notes and descriptions of larvae and pupae from Kenya, Uganda, Madagascar and Seychelles [182–191]. It is important to note that Ms. van Someren probably made most of her own line drawings, since some of the figures included in the first descriptions have her hand-written initials [182, 184]. Moreover, some of her publications contained specific edits to the identification keys available at the time, mostly keys originally provided by George Hopkins (1936, 1946) and F. W. Edwards (1941), and her descriptions of *Megharhinus* and *Eretmapodites* species from 1946 and 1949 include identification keys to the species of this genera [183, 188].

In other relevant work, Ms. van Someren and co-authors also provided detailed biological information about many mosquito species. One publication includes a list of all mosquitoes from the Kenya coast at the time, including their information on their occurrence, behavior and habitat [192]. Ms. van Someren and collaborators also described the abundance and behavior of mosquitoes after 2.5 years of observations and collections in two villages of the Kenya coast [193]. In yet another example, she listed the 156 species present in Tanganyika (currently part of Tanzania) and provided their distribution according to zoogeographic and climatic regions [194].

Surprisingly, there seem to be little to no formal descriptions published by Ms. van Someren in the early 1940s. There is, however, at least one publication on mosquitoes from British Somaliland (Somalia) authored by G. R. Cunningham van Someren, her husband (ornithologist) [195]. Here, the author mentions that the notes are based on a collection of mosquitoes that he and Dr. D. G. MacInnes made in 1942, and that he and Mrs. E. C. C. van Someren made the identifications. It is possible that she contributed to similar notes and descriptions published by others early in her career, although the precise identification of mosquito species may not have been considered significant enough to warrant co-authorship at the time.

Overall, the Systematic Catalogue of Culicidae compiled and maintained by the Walter Reed Biosystematics Unit lists at least 33 species and three subspecies in eight genera (*Aedes*,* Anopheles*,* Culex*,* Eretmapodites*,* Mimomyia*,* Orthopodomyia*,* Toxorhynchites* and *Uroanotaenia*) described by Ms. van Someren [196]. Also, at least two species, one subspecies and one subgenus (described as genus *Vansomereni*) have been dedicated to her. In recognition of her extraordinary work and the significance of her contributions, in 1974 Ms. van Someren was granted a Doctor of Technology *honoris causa* from Brunel University, UK (documents provided by Noel van Someren) and was appointed Member of the Order of the British Empire (MOBE) as part of Queen Elizabeth II’s Birthday Honors [197].

According to official sources, Ms. Ellinor C. C. van Someren died in Nairobi in 1988 [198].

## Brouria Feldman-Muhsam

Brouria Feldman-Muhsam was born in Jerusalem, Palestine (currently Israel) in 1916 [199]. Although her doctoral studies and career were also based in Jerusalem, she lived in Switzerland while obtaining her B.S in biology (Licence ès Sciences) from the University of Geneva, which she completed in 1937 [199–201]. While in Geneva, she met and married Helmut V. Muhsam, a physics doctoral student who had a faculty appointment at the Hebrew University of Jerusalem [199, 202]. Dr. Feldman-Muhsam returned to Jerusalem with her husband and obtained her Ph.D. in Medical Entomology from the Hebrew University of Jerusalem in 1942. In 1944, she began working as a research associate at the same university, in the Department of Parasitology. She was named head of the Department of Medical Entomology in 1968 and obtained the status of Professor of Parasitology at the Hebrew University of Jerusalem in 1978 [200]. In addition, she was a guest lecturer in the Institute of Acarology of the University of Maryland (1955–1959), a research associate in the Department of Entomology and Parasitology, University of California (1958) and a member of the Food and Agriculture Organization (FAO) expert panel on tick-borne diseases of livestock (1961–1962) (Kosta Y. Mumcuoglu, personal communication).

Dr. Feldman-Muhsam was recognized for her work on Acari, mostly ticks. Interestingly, some of her first studies were on the effects of irradiation on mosquito immature stages and the biology of flies [203–206]. Later, she began investigating tick behavior, specifically in *Hyalomma savignyi*, which was made possible with support from a fellowship of the International Association of University Women [207, 208]. Throughout her productive career (she published more than 100 scientific papers), some of her most notable work focused on tick ecology, physiology and mating behavior. For example, Dr. Feldman-Muhsam and collaborators described accessory glands of Gene’s organ in ixodid ticks and identified the porous areas as their outlets [209, 210], studied the formation of spermatophore and the transfer of sperm [211–213], analyzed the composition of male salivary secretions during mating [214] and made detailed observations on the copulation process of ixodid ticks [215], among many other contributions.

In addition to studying the physiology and ecology of ticks, Dr. Feldman-Muhsam was also an extraordinary tick and mite taxonomist. In this regard, her series of revisions on the genus *Hyalomma* is good example of her vast knowledge [216, 217]. She also made several original descriptions, including those on the genus and first species of *Stigmacarus* and on at least eight species of Acari in the genera *Haemaphysalis*, *Hyalomma*, *Rhipicephalus*, *Podapolipus*,* Tetrapolipus* [218], as well as on the endosymbiont genus *Adlerocystis* (with two species descriptions) [219]. At least four species have been named in her honor: *Haemaphysalis muhsami*, *Rhipicephalus muhsamae*, *Eutrombidium feldmanmuhsamae* and *Kurosapolipus feldmanmuhsamae* [220–223].

Dr. Feldman-Muhsam died in Jerusalem in 2008, leaving a legacy for future generations (Kosta Y. Mumcuoglu, personal communication).

## Elizabeth Nesta Marks

Elizabeth Nesta Marks was born in 1918 in Dublin, Ireland [224]. She did her primary schooling at St. John’s Cathedral Day School and then went on to boarding school for 4 years at the Glennie Memorial School, Toowoomba. In 1935, she enrolled at the University of Queensland (UQ) and 4 years later (1939) graduated with honors with a B.S. in Zoology. She specialized in entomology, with particular emphasis on insects of medical and veterinary importance [224]. At UQ, Dr. Marks was supervised by Dr. Ronald Hamlyn-Harris, an entomologist of the city of Brisbane and pioneer in biological control of mosquitoes in Australia, with whom she published the description of an anopheline larva [225]. In 1939, she started working as Assistant Curator at the Pathology Museum of the UQ Medical School [224].

Between 1942 and 1944 in Australia, there was an active push to study mosquito-borne diseases, mainly because of the exposure of Australian troops to diseases in malarious areas of New Guinea, a dengue fever epidemic in Brisbane and a malaria epidemic in Cairns [224]. This epidemiological scenario prompted the Queensland government to create Malaria Control Units and a Mosquito Control Committee (MCC). In 1943, Dr. Marks transferred to the UQ Department of Entomology as the MCC’s Graduate Research Assistant, which provided her with the economic support to study for 6 weeks at the British Museum of Natural History [224]. She subsequently studied for 2 years at Cambridge, UK where she successfully completed her Ph.D. requirements in insect physiology. In 1951 she returned to Queensland to continue her mosquito research [226]. The MCC was closed in 1973, and she was appointed Principal Entomologist at the Queensland Institute of Medical Research. Dr. Marks retired in 1983 and was awarded a Honorary Research Fellow [224].

Dr. Marks was a distinguished medical entomologist, who primarily specialized in mosquito taxonomy. In 1952, in collaboration with Kenneth Knight, she updated the taxonomic checklist of the subgenus *Finlaya*, genus *Aedes *in which they describe adults (male and female) and larva, create a taxonomic key and comment on the distribution and relationships of this subgenus [227]. Dr. Marks subsequently published an article in which she reviewed the *Aedes scutellaris* supgroup, emphasizing the variation in *Aedes pseudoscutellaris* (Theobald), mainly distributed in the Australasian and the eastern part of the Oriental region [228]. She also described other mosquitoes of the subgenera *Pseudoskusea* and *Neoculex* from the Australasian region [229], as well as a new species of *Anopheles* from Queensland, which she named *Anopheles colledgei* after W. R. College, an early Queensland student of mosquitoes [230]. In addition, Dr. Marks described a new species in the genus *Aedes * (*Finlaya*) from northern Australia, which was named *Aedes britteni* [231]. In 1960, due to the lack of evidence that the Australasian mosquito specimens in the Paris Museum (described by P-J-M. Macquart in 1850) were ever revised by subsequent entomologists, Dr. Marks and other colleagues compared type specimens in the museum with the Australian species, which resulted in the discovery of new synonyms and the description of *Aedes* (*Ochlerotatus*)* subalbirostris* as a new species from New Zealand [232]. In 1977, she described another new species in the genus *Aedes*: *Aedes* (*Macleaya*)* stoneorum* from Queensland [233]. Including the above-mentioned studies and many others, Dr. Marks alone or jointly described 38 new mosquito species [224]. In addition to her work on mosquito taxonomy, she also participated in studies on arthropod-borne viruses in Queensland, mainly by characterizing and isolating virus strains from wild-caught mosquitoes [102].

Dr. Marks received several awards and honors for her remarkable contributions to medical entomology, especially regarding mosquitoes. These included her appointment as Honorary Research Associate of the Bernice P. Bishop Museum (1974) and life memberships in the Queensland Naturalist’s Club, the Royal Society of Queensland and the Australian Entomological Society. Moreover, she was awarded the Australian Natural History Medallion in 1981 from the Field Naturalists’ Club of Victoria, and jointly with John Reid in 1986, she was given the Belkin Award of the American Mosquito Control Association for outstanding contributions in systematics [224]. In 1990, she was appointed Officer of the Order of Australia in the General Division, and in 1999 she received the Queensland Natural History Award [226].

Dr. Elizabeth Nesta Marks died in 2002 [226].

## Ana Esther Hoffmann Mendizábal

Ana Esther Hoffmann Mendizábal, also known as “Anita” Hoffmann, was born in Puebla, Mexico in 1919 [234]. Her father, Carlos Cristian Hoffmann, was a German entomologist and researcher who lived in Mexico and helped establish the Institute of Biology at the Universidad Nacional Autónoma de México (UNAM) [235]. He introduced Dr. Hoffmann to biological sciences and entomology at an early age, as she used to accompany him and his colleagues on fieldwork expeditions around Mexico [235]. She obtained her Master’s and D.Sc. in Biology at UNAM in 1941 and 1965, respectively [235]. At the time of receiving her Master’s degree, she began working at the Institute of Biology where 1 year later she became a research assistant. In 1944, she accepted an offer from the Institute of Public Health and Tropical Diseases of Mexico to work there on the physiology and reproduction of ticks infected with American spotted fever group rickettsiae [235]. As part of her education, she specialized in acarology after completing an internship at Duke University (Durham, NC, USA) and received intensive training with mite collections at the Smithsonian Museum. When she returned to México, Dr. Hoffmann continued studying the families of Acari, especially Trombiculidae, which was the group she had focused on in her doctoral thesis [235]. In 1965, she started to teach the first acarology course for graduate students and founded the first laboratory of acarology in Latin America, of which she was the head until her retirement. In 1974, Dr. Hoffmann was invited to teach zoology III (arthropods) at UNAM, and in 1975 she obtained a full-time Professor position at this university [235]. Dr. Hoffmann opened the second acarology laboratory in Mexico at UNAM, and since 1997 this laboratory bears her name as a tribute to her work [235].

Dr. Hoffmann was a pioneer in the study of mites and arachnids in Mexico, although she worked on a variety of topics in the fields of parasitology and entomology. She wrote over 130 articles and 10 books, including *Animales desconocidos: relatos acarológicos* (*Unknown animals: acarological tales*) (1988), *El maravilloso mundo de los arácnidos* (*The wonderful world of mites*) (1993) and *Biodiversidad de ácaros en México* (*Biodiversity of mites in Mexico*) (2000) [234, 235]. In this last book, 2343 specimens were registered, which made it a national catalog for mite research [235, 236]. Dr. Hoffmann also described approximately 60 taxa of mites; for example, a new genus and species of the Family Laepidae (*Chapalania cifuentesi*) and a new species of eriophyid mite *(Acalitus santibanezi*) [237–239]. Her work also involved the classification and description of ectoparasites of different mammals [240, 241], parasite-host relationships and various other Mexican tick species [235, 242]. Along with several colleagues, she also did research on human scabies [235, 243].

Throughout her career of 50 years, Dr. Hoffmann collected over 100,000 mites, 400 spiders, specimens from 13 families of diplopods and chilopods and various other ectoparasites. She donated this collection to the Institute of Biology of UNAM, which became the largest collection of mites in México, and incorporated it into the World’s Index of Acarology collections [234]. She also taught courses and held conferences on tick biology and control in different Mexican national institutions. Under her guidance, more than 30 Licentiate, four Masters and 12 Doctorate theses were produced [235].

In recognition of her distinguished career and contributions to her field, around 40 species have been named in her honor [235]. She was also awarded the National University Award (1990), the Diploma of Academic Merit from the Colegio de Biólogos de México (Mexican Board of Biologists) (1997), the title of Distinguished Researcher of the National Polytechnic Institute (IPN) and the Medal of College Merit, and was recognized as an emeritus researcher of the National Researchers System (1984) and Emeritus Professor of UNAM (2001). In addition, she was admitted as an honorary member of the Veterinary Parasitology Society (1974), the Biological Sciences Academy Society of the Universidad Autónoma de Nayarit (1983), the Mexican Zoology Society (1982), the Mexican Entomology Society (1995) and the Mexican Parasitology Society (1987) [235, 244].

Dr. Hoffmann worked and carried out research for over 60 years and died in Cuernavaca, Morelos, in 2007 [245].

## Ernestine Hogan Basham Thurman

Ernestine H. B. Thurman was born Ernestine Hogan Basham in Atkins, Arkansas, USA, in 1920. She attended elementary and secondary school in Atkins, and in 1944, she earned a B.S. from the University of the Ozarks (Clarksville, AR) [246, 247]. Dr. Thurman began her path in the study of biology even before graduating, serving at a local high school as the head of the Biology Department. Her career as an entomologist began when she acquired a position in the Malaria Control in War Areas program, ultimately leading the Bureau of Vector Control’s mosquito identification unit in Turlock, California [27]. It was there that she met and married Deed C. Thurman, who she accompanied on a journey to northern Thailand, both as commissioned US Public Health Service (USPHS) officers. Dr. Thurman was the first woman entomologist to be assigned to position [248].

Following the death of her husband, Dr. Thurman went back to the USA where she began working at the National Institutes of Health’s Microbiology Institute in Bethesda, Maryland. She subsequently obtained her Ph.D. in entomology from the University of Maryland, studying mosquitoes of Northern Thailand. After she moved to New Orleans and married Clyde Swartzwelder, she worked as an executive secretary for the Study Section on Tropical Medicine, Division of Research Grants of the USPHS. Once she retired from USPHS as Captain, she joined the faculty of the Louisiana State University Medical Center as a professor in pathology. Her last career steps took place when she resigned from that position and dedicated her later years to be a research fellow at the Center for the Study of Women at Tulane University, where she fought to overcome the barriers blocking the advancement of women in science [27, 246, 248].

Dr. Thurman’s work reflects a lifelong interest in mosquitoes and vector control [249–255]. Of note, she wrote the biography of Robert Evans Snodgrass, one of the most momentous American entomologists [256]. Before her tour in Thailand, which took place from 1951 to 1953, she authored and co-authored several scientific publications, mainly on ecological surveys, collections and descriptions of culicid mosquito specimens in the states of Florida and California. For example, she described the females, males, pupae and larvae of *Culex* (*Melanoconion*)* mulrennani* and *Aedes bicristatus*, both new species found in Florida and California, respectively [255, 257]. She also worked on the description of taxonomic characters in *Culiseta* larvae, which enabled the identification and differentiation of four species from California [254].

Undoubtedly, one of the most important contributions of Dr. Thurman was her participation in the founding of the Thai-American malaria control program. The program carried out operations that included the collection and study of mosquitoes, house-spraying with DDT, surveys, anti-malaria vigilance, training of medical officers and technical assistants and malaria treatment and public information [253]. The results of this initiative led to a vast knowledge on the mosquito fauna of the region, which was valuable information for the study and control of several mosquito-borne diseases. After being successful at reducing malaria cases, this program also served as a model for subsequent mosquito control programs in other countries. This initiative was beneficial not only at scientific and public health levels but also at the cultural level, since it was able to advance the establishment of relations among different countries and cultures, an aspect that can be an obstacle for implementing mosquito control measures [253]. Dr. Thurman’s honors include the National Defense Service Medal (1954, awarded 1963) [246].

Before Dr. Thurman died in January of 1987, in Virginia, she had influenced the field of entomology as a woman activist since she constantly expressed the need for women to be educated and more involved in science [248].

## Sister Monica Asman

Monica Asman was born in Germany in 1920, but moved to Los Angeles, USA, with her family when she was a child [258]. She became a member of the Sisters of St. Francis in 1940, and from 1944 to 1962 she was a teacher at different schools staffed by the Franciscan Order [258, 259]. In 1966, she received a Ph.D. in mosquito genetics from the University of Notre Dame (Notre Dame, IN, USA), which focused on chromosomal translocations of *Aedes aegypti* [260]. Of note, she was one of only eight women among 52 men who received this type of degree from that institution that year [259]. That same year (1966) she started working as an instructor in the Biology Department at Santa Clara University, where she remained until 1971; in 1968 she also joined the faculty of the University of California Berkeley as an Associate Research Entomologist, where she worked for almost 20 years [258–260]. After retiring from Berkeley, she opened a center for poor people in Redwood City, California [258].

As an expert in mosquito genetics, Dr. Asman produced more than 50 publications in several journals [259]. While working at Berkeley, she did research on *Culex tarsalis*, which plays a major role as a vector of St. Louis encephalitis and equine encephalomyelitis in the western USA. Her investigations focused on using chromosomal modifications as a tool for population control by introducing infertility in mosquito field populations (sterile male release method) or by making them less capable of transmitting disease [260–263]. Dr. Asman and collaborators irradiated male *Cx. tarsalis* with cobalt-60 to induce genetic alterations and determined the ideal radiation dose that induced approximately 95% mosquito sterility [262, 264]. Irradiated male mosquitoes were shown to be competitive when compared to non-irradiated males from both field and laboratory populations, determined by measuring the number of egg rafts produced by females exposed to both types of males [264]. She also participated in field releases of genetically altered (carrying sex-linked double heterozygous translocations) and radiosterilized *Cx. tarsalis* males, as well as studies on the mating ability of the latter mosquitoes in outdoor cages [265–267].

Other relevant contributions were the experiments carried out by Dr. Asman to determine the effects of ionizing radiation on the gonadal development of *Aedes aegypti* in different life-stages and studies on the genetics of *Aedes sierrensis*, as this species was also a candidate for the sterile male control method [268].

Dr. Monica Asman passed away in California in 2016 [259].

## Jane Brotherton Walker

Jane Brotherton Walker was born in Nairobi, Kenya in 1925 [269–271]. She spent part of her life in England, where she completed high school in 1944 and enrolled at the University of Liverpool. In 1948, she graduated with honors with a B.S. degree, and in 1959 she obtained her M.Sc. from that same university [271]. In 1949, she went back to Muguga, Kenya to work as a Research Officer in the East African Veterinary Research Organization, and in 1966, she accepted a position at the Veterinary Research Institute in Onderstepoort, South Africa [269]. She was awareded a D.Sc. by the University of the Witwatersrand in 1983 on her published works [270, 271]. During all this time, Dr. Walker continued to work at the Veterinary Research Institute until her official retirement in 1990, although she maintained honorary research activities until 1998, up to her decline in health (she had sequelae from contracting polio earlier in life) [269, 270].

Dr. Walker was a prominent tick expert, especially regarding the taxonomy of the genus *Rhipicephalus* and other species of Ixodidae found in Africa. Not surprisingly, she has been referred to as “the doyenne of African tick researchers” [269]. She published a total of 53 scientific publications and five books, either as a single author or co-author [269, 270]. In some of her first publications, she expanded on notes and made detailed descriptions of *Rhipicephalus pulchellus*,* R. pravus* and *R. humeralis* [272–274]. In subsequent publications, she included a series of descriptions and notes on the biology of tick species from several genera, but mostly *Rhipicephalus*, and it is relevant to note that she reared many of them in the laboratory [275–282]. Her notable knowledge of ticks is also exemplified in the books she published along with other tick experts, among which *The Genus Rhipicephalus* *(Acari, Ixodidae). A Guide to the Brown Ticks of the World* stands out as her most prominent contribution [283]. Dr. Walker also described 18 new tick species, most of them in the genus *Rhipicephalus* [218, 270, 271]. In these publications, the detailed descriptions and line drawings, which she mostly made herself, demonstrate her abilities and thoroughness [270, 271].

Dr. Walker’s contributions to the knowledge of ticks have been acknowledged in many forms by her colleagues. She was awarded several prestigious awards, including the 1988 Elsdon Dew Medal for “outstanding service rendered to Parasitology in Africa” (Parasitological Society of Southern Africa), the Agricultural Science and Technology Woman of the Year Award (1998) and the Theiler Memorial Trust Award in 1998 for “exceptional service rendered to Veterinary Science in Africa” [270, 271]. Also, several species of ticks have been named in her honor, such as *Ixodes walkerae*, *Argas walkerae*,* Rhipicephalus walkerae* and *Haemaphysalis walkerae* [284–287], as well as other mite species [223, 288, 289]. Moreover, her kind and collaborative spirit was evident and much appreciated; for example, Kaiser and Hoogstraal wrote “we dedicate this species to Miss Walker not only for this kindness but chiefly for her considerable contribution to knowledge of African ticks, some of which has been published but much more of which is available only in correspondence between Miss Walker and her many devoted colleagues” [287].

Dr. Jane B. Walker died at home in Pretoria, South Africa, in 2009, while enjoying tea with her colleague and mentee Ivan G. Horak [270, 271].

## Jadwiga Złotorzycka

Jadwiga Złotorzycka was born in 1926 in Warsaw, Poland, but grew up in Lviv, Ukraine [290, 291]. In her adolescence, she worked on a project about typhus with Dr. Rudolf Weigl, following which, in 1945, she was admitted to the University of Wroclaw, Faculty of Biological Sciences [290, 291], where she received the education that led her into entomology. She started working at the Museum of Natural History of the University of Wroclaw as a student and continued working there upon graduation; this work led her to pursue doctoral studies focused on bird lice of Poland. In 1972, Dr. Złotorzycka became head of the Department of General Parasitology at the University of Wroclaw, a position she held for 25 years [290–292].

Dr. Złotorzycka was an expert in the taxonomy of chewing lice (“mallophaga”), and her most important contributions focused on the biology, morphology and taxonomy of chewing lice species of birds [291–293]. However, she also studied the mallophaga of domestic mammals [290, 291, 294]. Among other topics, Dr. Złotorzycka investigated cuckoo wasps (Chrysididae), which were the subject of her Master’s thesis. In addition, she specifically focused on *Toxocara canis* and researched several aspects of parasites of dogs, apes, monkeys and mice [292, 293, 295–299].

Overall, Dr. Złotorzycka published close to 100 scientific papers, most of which were written in Polish, while others were published in German and English [298, 300, 301]. She also produced six identification guides, a catalog and a monograph on Polish chewing lice [292]. Moreover, she published at least 18 systemic reviews and expanded the zoological collection of the Museum of Natural History by depositing 717 specimens of chewing lice (124 species) in their collection [291–293]. In parallel, Dr. Złotorzycka described several new lice species as a single author, such as *Lanicanthus aequalis* and *Menacanthus verecundus*, or in collaboration with Dr. W. Eichler (*Gypsigogus novoannus*) [292].

To mention some of her many publications, in 1922, Dr. Złotorzycka collaborated with Ms. Maria Modrzejewska to record the presence and metric characters of lice (superfamily Ischnocera) collected from birds (order Procellariiformes) in islands of the Antarctic region of South Georgia [302]. Together they compiled one of the first records of lice in that region and found differences in the morphometric characters between the specimens collected and the registered data [302]. In that same year, Dr. Złotorzycka and Dr. Elżbieta Lonc analyzed and compared some of the alternative models for the classification of booklice (Psocodea) [300]; in this article they discussed the possible subjectivity in classification methods and the complexity of including aspects such as evolution, fossil registry and morphology in the classification of organisms. In addition to her scientific contributions, she wrote biographies of other scientists, including Abigniew Jara, Janina Janiszewska, Wolfdietrich Eichler, Michail Jakovlevic and Zbigniew Kozar [303–307].

In recognition of Dr. Złotorzycka’s numerous contributions, several species have been named after her, such as *Pseudomenopon zlotorzyckae* (= *Pseudomenopon pilosum*) and the subspecies *Anatoecus icterodes zlotorzyckae* [292]. Likewise, other phthirapterists dedicated new taxonomic units to her, such as the genus *Zlotorzyckiella* Eichler, the species *Docophorulus zlotorzyckae* (= *Philopterus zlotorzyckae)* and the subspecies *Saemundssonia platygaster jadwigae* [290].

According to biographies that have been written about her, Dr. Jadwiga Złotorzycka died in 2002 [290–292, 301].

## Rachel Galun

Rachel Galun was born in Tel Aviv, Palestine (now Israel) in 1926. According to her curriculum vitae (kindly shared by Professor Kosta Y. Mumcuoglu of the Hebrew University-Hadassah Medical School), she studied biology and agronomy at the Hebrew University of Jerusalem where she received her M.Sc. Degree in 1947. In 1955, she completed her Ph.D. in insect physiology and medical entomology in the Department of Entomology of the University of Illinois (Champaign, IL, USA). Between 1948 and 1952, she worked as a medical entomologist in the Israel Defense Forces, and between 1956 and 1977, she was the head of the Department of Entomology at the Israel Institute for Biological Research. From 1965 to 1975, she taught medical entomology and insect physiology at the University of Tel Aviv, Haifa Medical School and Israel Institute of Technology (Technion) in Haifa. Dr. Galun was appointed as a professor in Zoology at the Hebrew University in 1977, where she was also the head of the Department of Zoology from 1978 to 1982. From 1985 and until her retirement, she was involved in the Department of Parasitology and served as the head of the Institute of Microbiology (1987–1993) at the Hebrew University-Hadassah Medical School in Jerusalem.

Dr. Galun conducted research on a variety of topics and published approximately 170 articles in peer-reviewed journals. She studied the physiology of respiration and hematophagy in insects and leeches [308] and the role of purinergic receptors in the taking of blood meals by hematophagous insects [309–312]. She also evaluated rearing methods, tested and applied radiation and sterile techniques for vector control in soft ticks [313–315] and tested different chemical attractants, pheromones and baits as lures [316, 317]. Her contributions were diverse and also included the study of the ecology and epidemiology of ticks, Mediterranean fruit fly, midges and mosquitoes [318–321], as well as research on the nutrition of mosquitoes and flies and maggot therapy [322, 323]. Although her work focused mainly on flies, mosquitoes and ticks, she also worked with crustaceans [324], hymenopterans [325], moths [326], biting midges [327], fleas [328] and human head and body lice [329].

In 1992, Dr. Galun received the McArthur distinguished visiting professor award from the Center for Insect Science, University of Arizona. Among other academic activities, she organized seminars at the WHO (Geneva, Switzerland), the International Atomic Energy Agency (Vienna, Austria) and the International Center of Insect Physiology and Ecology, Nairobi (Kenya) (curriculum vitae provided by K. Y. Mumcuoglu). She also participated and organized multiple workshops and conferences in different continents, sat on a number of editorial boards and served as consultant and expert advisor on topics related to vector biology and control.

At the time of publication of this review, Dr. Galun is alive and residing in Israel (K. Y. Mumcuoglu, personal communication).

## Natalia Aleksandrovna Filippova

Natalia A. Filippova was born in Moscow in 1930. Her university years began in 1947 at the Faculty of Biology and Soil Sciences of Moscow State University, but her interest in entomology led her to specialize in this field under the tutelage of professors Aleksei A. Zachvatkin, Evgenii S. Smirnov and Vladimir N. Beklemishev. Her graduate studies, including M.Sc. (1952) and Ph.D. (1955) degrees, were on the identification of immature stages of ticks of the genus *Ixodes*, and the morphology and taxonomy of Ixodidae [330].

In the same year of her thesis dissertation (1955), Dr. Eugene N. Pavlovsky, head of the Russian School of Parasitologists and director of the Zoological Institute of the Academy of Russian Sciences at the USSR, invited Dr. Filippova to study and review the taxonomy of argasid ticks of epidemiological and epizootic importance in the south of the USSR. This led to the publication of a monograph on argasids of the Palearctic Region, which constitutes one of the most representative and comprehensive studies on this group of arthropods [330, 331].

After her work with argasids, Dr. Filippova dedicated herself to researching the biodiversity of ixodid ticks in the taiga, focusing on the study of *Ixodes persulcatus* [332]*.* This work resulted in the proposal of the “persulcatus group,” including representative species such as *Ixodes ricinus*, *Ixodes nipponensis*, *Ixodes kashmiricus*, and *Ixodes kazakstan* [333]. In these studies, she applied the ontogenetic methodology to improve understanding of the systematic relationships between ixodid species [334, 335]. Given the relevance of her career, the United Nations Educational, Scientific and Cultural Organization (UNESCO) put forward Dr. Filippova as the editor-in-chief of a collective monograph dedicated to *Ixodes* ticks of the “persulcatus group” of the taiga, included in the "Man and the Biosphere program”. Dr. Filippova was the author of approximately 30% of the topics, which included chapters on systematics, evolution, identification keys, individual and geographical variability of the different stages of ticks, geographical ranges of species and ecological relationships with species of sympatric zones, among others [330].

Dr. Filippova’s comprehensive research of Ixodida included studies on the genera *Haemaphysalis*, *Dermacentor*, *Hyalomma* and *Rhipicephalus* [334, 336–338]. Interestingly, she was not completely in agreement with the inclusion of *Boophilus* into the genus *Rhipicephalus* [330]*.* At the end of her career, she published a third monograph titled* The fauna of Russia and neighboring countries* [330]. Her contributions in taxonomy also included the description of 11 new species of ticks: *Anomalohimalaya lotozkyi*, *Argas latus*,* Argas macrostigmatus*,* Argas tridentatus*, *Argas vulgaris*,* Dermacentor montanus*,* Dermacentor ushakovae*,* Ixodes ghilarovi*,* Ixodes sachalinensis*,* Ixodes stromi* and *Ixodes subterranus* [135].

Among other academic contributions, Dr. Filippova also studied the relationship between *Borrelia burdorferi*, the causal agent of Lyme disease, and species of the ticks of the “persulcatus group”, taking into consideratopm aspects of distribution, ecology and evolution of *B. burdoferi* [330, 331].

As recognition of the impact of her contributions in the field of Ixodida, in 1993 she received the highest honor awarded by the Russian Academy of Sciences, the E.N. Pavlovsky Gold Medal [339].

Dr. Filippova died in St. Petersburg in 2018 [340].

## María Dora Feliciangeli

María Dora Feliciangeli was born in 1940, in the town of Borgomanero, in northern Italy [341]. During WWII, she was brought up in Rieti, where she attended elementary and secondary school from 1945 to 1958. In 1965, she obtained the degree of Doctor of Biological Sciences (Dottore in Scienze Biologiche) from Sapienza University, Rome [341]. In 1963, during her studies at that same university, she married José Piñero, who was an academic at the University of Carabobo, Venezuela, where she would later become a teacher and researcher. Dr. Feliciangeli arrived in Venezuela in 1966. She first worked as a microbiologist and chief of the Ecology Service of the Rural Endemics Division, School of Malariology and Environmental Sanitation, and then in 1972 she began working at the University of Carabobo [341]. At first, she was an instructor/teacher in the Parasitology Department, but in 1976 she became Aggregate Professor, and ultimately acquired tenure. In 1977, she moved to the UK where she began her doctoral studies, traveling back and forth to Venezuela, as she had been appointed coordinator of the Parasitology Program in addition to research coordinator. She obtained her Ph.D. degree from the University of London in 1982 [341].

Dr. Feliciangeli’s research focuses on relevant public health topics, such as the epidemiology of Chagas disease and leishmaniasis, immunological and molecular diagnosis of leishmaniasis, vector–parasite interactions, parasite genetics, sand fly and triatomine biology, chemically active substances and their effects on vectors and species descriptions [342–353].

Dr. Feliciangeli’s contributions to medical entomology are extensive. To name a few of her relevant academic accomplishments, her team established the co-existence of two transmission cycles of American cutaneous leishmaniasis, with *Lutzomyia migonei* as the putative vector, and described the infection of domestic animal hosts by *Leishmania guyanensis* for the first time [353]. Moreover, she described a new phlebotomine species in the *Lutzomyia longipalpis* species complex, namely *Lutzomyia pseudolongipalpis* [343]. Subsequently, she evaluated the effect of infection with an autochthonous Venezuelan *Leishmania infantum* strain on the fecundity-fertility, survival and life expectancy of both *Lu. longipalpis* sensu lato and *Lu. pseudolongipalpis*, observing a detrimental effect in all parameters [342]. In addition, she assessed the putative role of *Rhodnius robustus* as a vector of *Trypanosoma cruzi* in western Venezuela, when *Rhodnius prolixus* is absent [349].

An extensive list of distinctions have been awarded to Dr. Feliciangeli, including various awards and certifications related to her field of study, as well as the title of International Woman of the Year (1999) awarded by the University of Cambridge for her contributions to medical entomology and parasitology [341]. She is also ranked as the University of Carabobo’s top researchers and considered to be one of the best medical entomologists in Latin America, with over 136 published papers. In 2008 and 2012 she was a member of the WHO/PAHO (Pan American Health Organization) Steering Committee for Chagas Disease and the Expert Committee of Entomological Phlebotomine Vigilance, respectively. It is also noteworthy that Dr. Feliciangeli was a member of many major scientific societies, such as the Royal Society of Tropical Medicine and Hygiene, the Royal Entomological Society of London and the Entomological Society of America, while she also presided the Venezuelan Parasitological Society [341]. Lastly, a specialized committee dedicated to the study of arthropods of medical importance within the Venezuelan Science Incubator was named after her [354].

After a lifetime of achievements in medical entomology and vector-borne diseases, Dr. María Dora Feliciangeli died in 2017, in the city of Maracay, Venezuela, at the age of 77, leaving the field to a new generation of professionals and trainees [341].

## María Cristina Ferro

María Cristina Ferro was born in 1947 in Ipiales, Colombia. She finished her B.S. in Microbiology at Universidad de los Andes, Bogotá, in 1969, and then joined the Entomology Group of the National Institute of Health of Colombia (INS), where she began to work and conduct research on leishmaniasis vectors [355]. During 1975 and 1976, she completed a M.Sc. degree in Medical Parasitology at the School of Hygiene and Tropical Medicine in the UK, working with xenodiagnosis of *Trypanosoma cruzi* [356]. Upon her return to Colombia, she resumed her work at INS and coordinated the Entomology Laboratory from 1994 until her retirement in 2005, but continued to be a consultant and advisor there until 2015. Under her supervision, this laboratory was designated as a category “A” laboratory and earned the "Group of Excellence" award by the Administrative Department of Science, Technology, and Innovation of Colombia (Colciencias) in 1995. In 2006, she joined the editorial committee of the Colombian scientific journal* Biomédica*, where she was involved in the review and evaluation of articles related to entomology [355].

Ms. Ferro's main research focused on the study of the vectors of leishmaniasis and Venezuelan equine encephalitis, but she also worked on triatomines, biting midges and the epidemiology of vector-borne diseases [357–359]. Focusing on the phlebotomines of Colombia, she studied the distribution, biology, and genetics of various species, and described three new species of *Lutzomyia* (*Lutzomyia torvida, Lutzomyia falcata* and *Lutzomyia tolimensis*) [360–362]. She also described isolates of *Leishmania* spp. and new viruses from sand flies, and carried out research on vector incrimination, vector capacity and environmental and ecological factors associated with the transmission of leishmaniasis [363–368]. In recognition of her many contributions to this field, a new species of *Lutzomyia* was named in her honor: *Lutzomyia ferroae* [360]. Regarding Venezuelan equine encephalitis, her studies focused on the identification, biology and ecology of the mosquito species identified as vectors, as well as on the genetics and life-cycles of the epizootic vectors in the laboratory and the epidemiological determinants of disease transmission [369]. In addition, she studied triatomine vectors of Chagas disease and, for her contributions in this topic, a new species of triatomine bug, *Belminus ferroae,* was named in her honor in 2007 [370–372]. She also made important contributions to the biology and ecology of the family Ceratopogonidae, specifically concerning the genus *Culicoides*, in which some species are considered potential vectors of different microorganisms [373].

Ms. Ferro’s scientific career covered multiple aspects. She was not only extremely productive academically, with her name as author of more than 100 published articles and several book chapters, but also served as an advisor of undergraduate and graduate students and actively participated in different national and international scientific events on tropical medicine, parasitology and entomology [355]. Likewise, she collaborated with national and international researchers with links to universities and research centers, such as the International Center for Medical Training and Research, the Center for Research in Microbiology and Tropical Parasitology of the Universidad de los Andes, Yale University, the University of Texas, the University of Florida and La Salle University, as well as with researchers with links to different health ministries of Colombia [355]. In 2007, the INS awarded her the distinction of “Emeritus Researcher,” in recognition of her investigations in the field of medical entomology. For her scientific and academic merits, she received other distinctions, such as the “Ernesto Osorno Mesa” Award for work presented at the Congress of the Colombian Entomology Society, recognition as Senior Researcher by Colciencias and the Honorable Mention awarded by Colciencias and Semana magazine in the category of “Medical and Health Sciences” of the Grand Prize for Life and Work of Emeritus Researchers of Colombia, in 2014 [355].

In a tribute to Ms. Ferro, following her death in Colombia in 2015, many researchers praised not only her academic excellence, but also her collaborative attitude and passion for learning and teaching [374]. She is recognized for her contributions in the development of science and innovation in Colombia, as well as for her collaboration in the structuring, development and consolidation of the National Network of Medical Entomology of her country [355].

## Honorable mention: Clara Louise Maass

Clara Louise Maass is included in this review as an honorable mention, despite not being a medical entomologist or working directly in this discipline, due to her historical contribution to the field as a volunteer in experiments related to the transmission of YF.

Ms. Clara Maass was born in the US state of New Jersey in 1876, in an immigrant family from Germany [375]. When she was 17 years old, she enrolled at the Christina Trefz Training School for Nurses at Newark German Hospital for a 2-year program; 3 years after completing her degree, she became a head nurse at that same hospital [376, 377]. In 1898–1899, she worked for 4 months as a contract nurse in the US Army during the Spanish-American War and served in the USA (Florida and Georgia) and Cuba (Santiago) [378]. Near the end of 1899, she traveled to the Philippines as a volunteer, where soldiers were suffering from dysentery, smallpox, typhoid and YF [375–377, 379].

In 1900, Ms. Maass applied for a call for nurses made by Dr. William Gorgas, the Chief Sanitary Officer of Havana, Cuba, and started to work as a nurse at Las Animas Hospital. Dr. Gorgas, and other members of the Sanitary Department decided to establish the Las Animas inoculation station at the hospital, with the aim to study vaccination as a measure to control YF. To this end, they used infected mosquitoes to induce mild disease among non-immune individuals [376, 380]. Between March and June of 1901, at the age of 25, Ms. Maass volunteered five times for experimental inoculations. Believing she lacked immunity, she was inoculated one more time on 14 August, using mosquitoes that had been infected by being exposed to a patient who had experienced a virulent form of the disease. Approximately 4 days later, Ms. Maass developed fever, chills and a headache. Unfortunately, her illness evolved quickly to a severe and intensely hemorrhagic one, which led to her death on 24 August 1901 [380]. Two more fatalities occurred, all resulting from the bites of mosquitoes infected from a single patient, after which the researchers concluded that these deaths were objectionable and the experimental inoculations at Las Animas Hospital ceased [375, 376, 380].

The Cuban and USA governments recognized Ms. Maass’s volunteer contributions to science and, in her remembrance, a plaque of her hangs on the walls of the Las Animas Hospital in Havana and in the Newark German Hospital, renamed as the Clara Maass Medical Center [381]. The latter hospital is home to a museum and an archive of documents that honor her memory [376]. In addition, in 1951 and 1976 the Cuban and US postal services, respectively, issued stamps commemorating her memory, and in 1976 she was introduced as a member in the American Nurses Association Hall of Fame. She is buried in Newark Fairmount Cemetery in New Jersey [377, 378, 381, 382].

Experiments on YF with human subjects ended after these three deaths, but these trials provided scientists and non-scientists with the opportunity to reflect upon the use of humans in research and, together with other events that occurred later in history, laid the foundations for good clinical practices and the creation of Institutional Review Boards. These YF experiments were also the first to formulate a typed informed consent form, in English and Spanish, and signed by the volunteers [376]. Some of the medical physicians involved in these experiments have been recognized for the role they played in understanding and controlling YF, but the contribution of volunteers who participated in these experiments to the field of medical entomology should be acknowledged and honored; ultimately, their deaths gave guidelines for patient treatments, invalidated the hypothesis that experimentally acquired YF was milder than naturally acquired disease, evidenced the risks of inducing immunity even under ‘controlled’ conditions and allowed further study of the disease and mosquito infection (intrinsic and extrinsic incubation periods, vector vertical transmission and effect of temperature on vectorial capacity) [380, 383].

## Conclusions

The women portrayed in this review have undoubtedly helped to lay the foundations of modern medical entomology. They exemplify the variety of specializations and arthropod groups in this field, such as taxonomy, physiology, vector control and epidemiology of the diseases caused by the pathogens they transmit. It is evident that a review such as this one is not complete and that there are probably many other women who could have been included. Also, it was not possible to portray representatives from every region of the world. In most countries, including those with a history of significant scientific development, such as India, China, Japan, Spain or Brazil, women have contributed to the advancement of science, but names in the history of medical entomology, in particular, seem less easy to track [384–391]. In this context, the current underrepresentation of women in some areas of science is determined by multiple and diverse upbringing, social, educational and labor-economic factors, which also manifest differently in each country [392, 393]. These historical and cultural contexts, in addition to the availability of digital past records, the language of publications and access to communication technologies, may complicate the search for biographical information and achievements of such women in medical entomology. This was the case for Vida Lester MacDonell and Carrie Collins Aaron, for whom information was incomplete or inaccessible despite their names being mentioned in other publications [27]. The task of identifying and acknowledging the role of all these women in history should be undertaken and encouraged. Nevertheless, the women included in the present document represent the determination and sacrifice of so many extraordinary women who, at the time, struggled to follow their aspirations in disciplines dominated by men, with some even losing their lives doing what they loved. There is no doubt that their work transcends to the present day, not only in their numerous scientific contributions but also in the paths they have opened for others to follow.

## Data Availability

Not applicable.

## Abbreviations

ASTMH: American Society of Tropical Medicine and Hygiene
B.S.: Bachelor of Science
Colciencias: Administrative Department of Science, Technology, and Innovation of Colombia
DDT: Dichlorodiphenyltrichloroethane
D.Sc.: Doctor of Science
FAO: Food and Agriculture Organization
INS: National Institute of Health of Colombia
IPN: National Polytechnic Institute
MCC: Mosquito Control Committee
PAHO: Pan American Health Organization
Ph.D.: Doctor of Philosophy
UNAM: Universidad Nacional Autónoma de México
UNESCO: United Nations Educational, Scientific and Cultural Organization
UQ: University of Queensland
USPHS: United States Public Health Service
USSR: Union of Soviet Socialist Republics
WWII: World War II
YF: Yellow fever
